# Verifying the accuracy of self-reported smoking behavior in female volunteer soldiers

**DOI:** 10.1038/s41598-023-29699-2

**Published:** 2023-03-01

**Authors:** Wei-Hung Chan, Ching-Huang Lai, Shu-Jia Huang, Chung-Chi Huang, Chung-Yu Lai, Yi-Chun Liu, Shiang-Huei Jiang, Shan-Ru Li, Ya-Mei Tzeng, Senyeong Kao, Yu-Tien Chang, Chia-Chao Wu, Chao-Yin Kuo, Kuang-Chen Hung, Yu-Lung Chiu

**Affiliations:** 1 School of Medicine, National Defense Medical Center, Taipei, 114 Taiwan; 2 Department of Anesthesiology, Tri-Service General Hospital, National Defense Medical Center, Taipei, 114 Taiwan; 3 School of Public Health, National Defense Medical Center, Taipei, 114 Taiwan; 4 Graduate Institute of Aerospace and Undersea Medicine, National Defense Medical Center, Taipei, 114 Taiwan; 5 Graduate Institute of Medical Sciences, National Defense Medical Center, Taipei, 114 Taiwan; 6 Graduate Institute of Life Sciences, National Defense Medical Center, Taipei, 114 Taiwan; 7 Department of Internal Medicine, Tri-Service General Hospital, Taipei, 114 Taiwan; 8 Department of Otolaryngology-Head and Neck Surgery, Tri-Service General Hospital, Taipei, 114 Taiwan; 9 Institute of Cognitive Neuroscience, National Central University, Taoyuan, 320 Taiwan; 10 Division of Neurosurgery, Department of Surgery, Taichung Army Force General Hospital, Taichung, 411 Taiwan; 11 Department of Surgery, National Defense Medical Center, Taipei, 114 Taiwan; 12 General Education Center, College of Humanities and General Education, Central Taiwan University of Science and Technology, Taichung, 406 Taiwan; 13 Department of Healthcare Administration, College of Management, Central Taiwan University of Science and Technology, Taichung, 406 Taiwan

**Keywords:** Health care, Health occupations

## Abstract

Smoking rates in the military are evaluated through questionnaire surveying. Because the accurate identification of smokers facilitates the provision of smoking cessation services, this study conducted urine cotinine concentration testing to verify the accuracy of self-reported smoking behavior by female volunteer soldiers and analyzed the effects of second-hand smoking on urine cotinine concentrations. This study is a cross-sectional study conducted using purposive sampling on female volunteer soldiers receiving training at the Taichung Recruit Training Center in May 2014. This study simultaneously collected questionnaires and urine samples, and urine samples were analyzed with an enzyme-linked immunosorbent assay. The self-reported smoking rate of female volunteer soldiers was 19.3%, whereas the smoking rate as determined by urine cotinine concentration testing was 26.3%, indicating an overall underestimation of 7.0%. Chi-square (χ^2^) goodness of fit test results indicated that the distribution of self-reported smoking behaviors and that verified from urine cotinine concentration testing were significantly different. The sensitivity of self-reported smoking behavior was 66.7% with a specificity of 97.6%. There was no significant association between second-hand smoking and urine cotinine concentrations. Questionnaire survey self-reporting methods could underestimate the smoking behavior of female volunteer soldiers and routine testing with biochemical verification is necessary.

## Introduction

Smoking is not only associated with several diseases, such as cancer, chronic lung disease, and heart disease, but it also causes negative health effects unique to females, such as infertility, stillbirths, low infant birth weight, greater perinatal mortality, and cervical cancer^1^. As female individuals have lower smoking rates compared to their male counterparts^2^, they are often overlooked by antismoking policies. However, smoking cessation is more difficult for female than for male, because of various concerns such as weight gain, hormonal fluctuations during the menstrual cycle, and their greater susceptibility to stress^3^, which lead to lower smoking cessation effectiveness^4,5^.

According to 2020 Adult Smoking Behavior Survey in Taiwan, the smoking rates of male and female are 14.3% and 7.5%, respectively, in the age of 21 to 25 group^6^. However, the male and female soldiers' smoking rates are 36.2% and 11.9% in Taiwan^7^, both higher than that of the age 21 to 25 group.

To effectively provide tobacco control intervention programs and accurately find the first step for providing smokers with methods of smoking cessation, many of those seeking to evaluate smoking behavior use questionnaire surveys because of its speed, low-cost, and ability to collect large volumes of information. However, a literature review of 54 previous studies indicated that self-reported smoking rates generally lead to average underestimation of 4.8% to 9.4%^8^. The underestimation of smoking rates varies according to the research subject, and past studies have found that the underestimation of self-reported smoking rates is more severe for female in Asian countries compared with male^9,10^. By contrast, there was no significant difference in the underreporting rates between the two sexes in Western countries^11^. This result may be because of the greater degree of societal disapproval towards female smoking in Asian countries, particularly among pregnant female or patients with smoking-related diseases, who may hide their smoking behaviors to meet societal expectations^12,13^.

Compared to questionnaire surveys, biomarkers are a more objective indicator of smoking. Cotinine is an alkaloid present in tobacco that can be detected in both blood and urine, and is typically used as a biomarker for tobacco exposure^14,15^. Testing of cotinine in urine is noninvasive and is highly correlated with cotinine in blood serum and plasma^16^, and is often used as a method for verifying smoking behaviors^9–11,17^. The Taiwanese military uses self-reported questionnaire surveys to monitor smoking rates^18^, with past surveys indicating a smoking rate of 12.1% among female volunteers^19^, or 3.3 times that of the female in the same age group^6^. Clearly, smoking is a significant health problem for female volunteer soldiers. A male conscripts study reveals that the conscripts’ self-reported and cotinine-validated smoking rates (30.9% vs. 31.9%) had a high level of consistency^20^, but past studies have indicated that female Asian smokers tend to conceal smoking behavior^10^. There remains few studies on female Asian soldiers and further research is needed. As such, this study conducted urine cotinine testing to verify the accuracy of self-reported smoking behavior by female volunteer soldiers. In addition, because second-hand smoke exposure may affect the cotinine concentration of nonsmokers^21^, this study also analyzed the effects of such exposure on urine cotinine concentrations.

## Methods

### Study design and participants

This study is a cross-sectional study examining female volunteer soldiers who were aged at least 18 years old through a purposive sample of female volunteers trained at the Taichung Recruit Training Center in May 2014. Six participants did not provide a urine sample and two did not complete the questionnaire. The response rate was 95.0%. Written informed consent was obtained from all participants. The experiment design and procedures of this study were reviewed and approved by the Institutional Review Board of the Tri-Service General Hospital, National Defense Medical Center and performed according to the guidelines of the Declaration of Helsinki (No. 2-103-05-012). The day before new recruit training was to conclude, experiment personnel explained the purpose of the study, obtained consent from the research participants, distributed the questionnaires, and collected urine samples.

### Procedure and instruments

This study referenced the design of past experimental surveys^21^. To assess smoking behavior, this survey inquired “Do you currently have a habit of smoking?” with options comprising “currently smoking”, “already quit smoking”, and “never smoked”. Those indicating that they have quit smoking or had never smoked were considered to have no smoking behavior. Second-hand smoke exposure was assessed by asking research participants “Has anyone smoked in front of you within the last 30 days?” with options comprising “yes” and “no”.

Urine samples were collected on the same day as the questionnaire surveys. The urine of each research participant was collected in 500 mL wide-mouth bottle containers, and then placed in – 4 °C ice buckets for storage. Subsequently, the samples were transported to a laboratory within 4 hours for storage in a refrigerator at – 20 °C. Within one week of collection, urine cotinine concentration analysis was conducted with enzyme-linked immunosorbent assays (ELISA) kits (Calbiotech Co., Spring Valley, CA, USA), with participants who exhibited urine cotinine concentrations above 50 ng/mL considered to be smokers and who exhibited urine cotinine concentrations under 50 ng/mL considered to be nonsmokers (Society for Research on Nicotine and Tobacco Subcommittee on Biochemical Verification, 2002). The testing process can be found in our previous study^20^.

### Data analysis

Statistical analysis was conducted using IBM SPSS Statistics V.22.0 (IBM, Armonk, NY, USA), with the number of samples, percentage, mean, and standard deviation describing the distribution of the demographics of the research participants, smoking behavior, and exposure to second-hand smoke. A chi-square (χ^2^) goodness of fit test was used to analyze the association between self-reporting and urine cotinine concentration verification of smoking behavior, with the Kappa value representing the consistency between the two types of instruments. A Kappa value > 0.75 was considered to be excellent, a value between 0.4 and 0.75 represented a fair to good result, and a value < 0.4 represented a poor result^22^. In addition, sensitivity and specificity was used to analyze the accuracy of self-reported smoking behavior. The Mann–Whitney *U* test was used to analyze the relationship between self-reported smoking behavior and exposure to second-hand smoke with urine cotinine concentration. Finally, Fisher’s exact test and chi-square (χ^2^) testing was used to analyze the relationship between exposure to second-hand smoke and urine cotinine concentration groups.

## Results

### Characteristics of study population

This study surveyed a total of 120 female volunteer soldiers with an average age of 23.3. The majority, or 72 individuals (66.7%), had less than high school education, and 19 individuals (15.8%) reported exposure to second-hand smoking (Table 1).

**Table 1:** Female volunteer soldier demographics (N = 120).

Demographics	Mean ± SD	n (%)
Age	23.3 ± 3.6	
Education level		
High school and below		72 (66.7)
Technical college and above		36 (33.3)
Exposure to second-hand smoke		19 (15.8)

### Comparison urine cotinine concentration of self-reported smoking behavior and second-hand smoke exposure

Table 2 outlines the comparison between self-reported smoking behavior and urine cotinine concentration. Self-reported smokers exhibited significantly higher urine cotinine concentrations compared to non-smokers (363.4 ng/mL vs. 36.3 ng/mL, *p* < 0.001). The self-reported smoking rate was 19.3%. Using a urine cotinine concentration of 50 ng/mL as the cut-off point, those with results higher than 50 ng/mL were considered to be smokers and those with results lower than 50 ng/mL were considered to be nonsmokers. Thus, a total of 26.3% of participants were considered smokers with their urine cotinine concentration higher than 50 ng/mL. The chi-square (χ^2^) test comparing the self-reported and urine cotinine concentration groups statistically significant level of difference (*p* < 0.001), indicating that the distribution of self-reported smoking behavior did not match that of the smoking behavior as verified by urine cotinine concentration testing. The smoking behavior determined by self-reporting and urine cotinine concentration testing exhibited a Kappa coefficient of 0.703 with acceptable consistency^22^. The overall self-reported smoking behavior had a sensitivity of 66.7% and a specificity of 97.6%.

**Table 2:** Comparison of self-reported smoking behavior and urine cotinine concentration.

Self-reporting	Total n (%)	Cotinine (ng/mL) mean ± SD	*P-*value^a^	Urine cotinine concentration grouping		*P*-value^b^
				< 50 ng/mL n (%)	≥ 50 ng/mL n (%)	
Nonsmoker	92 (80.7)	36.3 ± 101.8	< 0.001	82 (71.9)	10 (8.8)	< 0.001
Smoker	22 (19.3)	363.4 ± 182.3		2 (1.8)	20 (17.5)	
Total	114 (100.0)			84 (73.7)	30 (26.3)	
					Kappa = 0.703 Sensitivity = 66.7% Specificity = 97.6%	

^a^Mann–Whitney *U* test; ^b^Chi-square (χ^2^) goodness of fit test.

The effects of second-hand smoke exposure on the urine cotinine concentrations of female volunteer soldiers were then categorized and compared, according to self-reported smoking behavior (Table 3). Half of self-reporting smokers indicated they had been exposed to second-hand smoke, whereas the other half said they had not. 92.4% of self-reporting nonsmokers stated they had not been exposed to second-hand smoke, while 7.6% said they had. The results indicated that for self-reported smokers, those exposed to second-hand smoke exhibited a median urine cotinine concentration of 417.6 ng/mL, whereas those reporting no exposure had a lower concentration of 352.0 ng/mL. There was no statistically significant difference in the urine cotinine concentrations between those exposed to second-hand smoke and those not exposed (*p* = 0.519). Similarly, there was no statistically significant difference in the urine cotinine concentrations between nonsmokers exposed to second-hand smoke and those not exposed (2.0 ng/mL for those with exposure vs. 2.8 ng/mL for those without exposure, *p* = 0.466). Using 50 ng/mL as the urine cotinine concentration cut-off point for determining smoking behavior, all self-reported smokers exposed to second-hand smoke were above the cut-off point and 81.8% of self-reported smokers not exposed to second-hand smoke were above the cut-off point. However, second-hand smoke exposure exhibited no significant association with urine cotinine concentration groupings (*p* = 0.476). Among the self-reported nonsmokers, a higher percentage of those exposed to second-hand smoke exhibited urine cotinine concentrations categorizing them as smokers compared with those not exposed, but the results did not reach a level of statistical significance (28.6% for those exposed vs. 9.4% for those not exposed, *p* = 0.350).

**Table 3:** Comparison of female volunteer soldiers’ second-hand smoke exposure and urine cotinine concentration.

Self-reporting	Total n (%)	Cotinine (ng/mL) median	*P-*value^a^	Urine cotinine concentration grouping		*P*-value
				< 50 ng/mL n (%)	≥ 50 ng/mL n (%)	
Smoker			0.519			0.476^b^
Exposure to second-hand smoke	11 (50.0)	417.6		0 (0)	11 (100.0)	
No exposure to second-hand smoke	11 (50.0)	352.0		2 (18.2)	9 (81.8)	
Nonsmoker			0.466			0.350^c^
Exposure to second-hand smoke	7 (7.6)	2.0		5 (71.4)	2 (28.6)	
No exposure to second-hand smoke	85 (92.4)	2.8		77 (90.6)	8 (9.4)	

^a^Mann–Whitney *U* test; ^b^ Fisher’s exact test; ^c^ chi-square (χ^2^) test.

## Discussion

This study examined the verification of self-reported smoking behavior through urine cotinine concentration testing of female volunteer soldiers. The results indicated that the self-reported smoking rate was 19.3%, whereas the smoking rate based on urine cotinine concentration testing was 26.3%, with self-reporting underestimating the smoking rate by 7.0%. The results of chi-square (χ^2^) goodness of fit test revealed that the distribution of self-reported smoking behavior is different to that of smoking behavior verified through urine cotinine concentration testing, and the consistency of the two evaluation methods was only acceptable (Kappa = 0.703)^22^. Notably, the degree of underreporting is similar to that of South Korea. The studies verifying self-reported smoking behavior through urine cotinine concentration testing (50 ng/mL as the cut-off point) conducted by the 2008-2009 Korean National Health and Nutrition Examination Survey determined that the self-reported female smoking rate (6.6%) was lower than the actual smoking rate (14.5%) by 7.9%^10^. However, in Western countries, self-reported smoking behavior and smoking rates as verified through urine cotinine testing was consistent; this was demonstrated by the Canadian Health Measures Survey, which found that the self-reported female smoking rate was 18.6%, whereas the female smoking rate verified through urine cotinine testing was 19.0%: an underestimation of 0.4%^11^. Relatively, a male conscripts study in Taiwan show that the self-reported smoking rate was 30.9%, whereas the cotinine-validated smoking rate was 31.9% (≥ 100 ng/mL): an underestimation of 1.0%^20^. Another study in Korean, absolute differences between cotinine-verified and self-reported smoking rates was 5.0%^9^. These results indicated a smaller underestimation than the results of the present study. As demonstrated, the self-reported smoking rates of Asian female exhibit markedly more severe underreporting compared with Western female. Although this study was conducted in 2014, the smoking rate among Taiwanese females only decreased by 0.6% in 2010 compared to 2014^23^, and there has not been a significant change in social culture, and there is no significant difference in the recruitment methods and numbers of female soldiers in Taiwan through 2022. Therefore, the underreporting of smoking among female soldiers still warrants attention and routine testing with biochemical verification is necessary.

A review of ten studies that had used urine cotinine concentration to confirm self-reported smoking behavior indicated that the average sensitivity of self-reported smoking behavior is 75%^8^, with the degree of sensitivity differing according to the research participant. If female are used as the comparison group, the sensitivity of self-reported smoking behavior by female volunteer soldiers in this study was 66.7%; this is higher than that of South Korean female (44.0%)^10^ and pregnant female (12.5%)^13^, but lower than that of Canadian female (91.2%)^11^. However, a male conscripts study in Taiwan adopted the UC concentration of 100 ng/mL as the baseline to identify smokers, the overall sensitivity was 92.9%^20^. The lower sensitivity exhibited by the self-reported smoking behavior of female volunteer soldiers in this study may be because of errors in self-awareness: for example, it is inconvenient for soldiers to smoke during the training period but they may still smoke off-duty, or they may believe that they have already quit, but continue to exhibit urine cotinine concentrations above the cut-off point (50 ng/mL). Furthermore, the results of this study considered those with higher urine cotinine concentrations (≥ 50 ng/mL) to be smokers, but of the ten individuals who self-reported as non-smokers, seven individuals reported that they had previously smoked and thus had higher urine cotinine concentrations. In addition, the higher rate of concealing smoking behavior by female in Asian countries than in Western countries may be because of cultural differences. Asian countries are often more conservative and generally believe that smoking is a male right; conversely, female who smoke may face greater social pressures and are therefore more likely to conceal smoking behavior^24^.

This study further analyzed the effects of second-hand smoke exposure on urine cotinine concentrations, and determined that second-hand smoke exposure did not significantly increase the urine cotinine concentration of smokers or nonsmokers. This result was similar to the military service and basic military training conscripts research^20^. Prior study indicated that smoking in front of participants and perceived frequency of SHS exposure in past 7 days were related to urinary cotinine among nonsmoking pregnant female^25^. Because there were restrictions on when and where you can smoke at the Recruit Training Center, the non-significant result in this study may be attributable to the similar second-hand smoke exposure of smokers and non-smokers in military. According to the urine testing that confirmed smoking behavior, self-reported nonsmokers exposed to second-hand smoke exhibited higher rates of incorrect reporting of smoking behavior than those not exposed to second-hand smoke (28.6% vs. 9.4%); this is similar to the findings of past newly diagnosed cancer patients’ study, although the results did not reach a level of statistical significance^26^. However, a Korea research found nonsmokers' underreporting of their smoking status was positively associated with secondhand smoke^27^. The non-significant finding in this study could be owing to the female volunteer soldiers who had served in the military for a long time everyday being exposed to nearly the same background second-hand smoke. Despite exposure to second-hand smoke increasing urine cotinine concentrations, the concentration typically does not exceed 20 ng/mL^28^. Therefore, exposure to second-hand smoke did not cause the urine cotinine concentrations of nonsmokers to exceed 50 ng/mL, nor result in incorrect reporting.

The female volunteer soldiers' self-reported smoking behavior and urine cotinine tests were found to be lower consistent in this study (Kappa = 0.703) than the results of the following studies^11,20,29^. The military service and basic military training conscripts' self-reported smoking behavior was shown to be highly consistent with their urinary cotinine levels (Kappa = 0.918)^20^. Within the Malaysian cohort project, urinary cotinine concentration was found to be correlated with self-reported smoking status, indicating that the self-reported smoking questionnaire is a reliable tool for assessing the tobacco use (Kappa = 0.822)^29^. Canadian Health Measures Survey also found self-reported smoking status can be used to calculate realistic smoking rate among Canadians (Kappa = 0.950)^11^.

Although the results of this study showed inconsistencies between self-reported smoking behavior and urine cotinine concentration testing by female volunteer soldiers, some research limitations exist. First, this study conducted purposive sampling, and therefore is unable to represent the entire female volunteer soldier population. Second, determining the urine cotinine concentration cut-off point for smokers affects the sensitivity and specificity of self-reporting and cotinine-verified smoking rates; furthermore, the cut-off point will differ based on the racial/ethnic groups studied, e.g., 50, 100, or 250 ng/mL, etc.^9,10,20,21,30^. This study referred to the study by the Society for Research on Nicotine and Tobacco and used 50 ng/mL as the cut-off point as set by related studies conducted in South Korea^10,31^ that examined and compared smokers. Third, this study did not collect the time and location of exposure to second-hand smoke; however, because the daily routines of female volunteer soldiers are identical during their training period, exposure to second-hand smoking is likely to have occurred in similar locations and times. Thus, the effects of these factors on urine cotinine concentrations in this instance can be disregarded. Fourth, the effect of diet on urine cotinine concentrations has been reported to be between 0.6 and 6.2 ng/mL^32^. Because female volunteer soldiers have the same diet during the training period, the effects of diet on urine cotinine concentrations can be disregarded in this instance.

## Conclusions

This study found that the self-reported smoking behavior of female volunteer soldiers, and their smoking behavior as verified by urine cotinine testing, was not consistent. Therefore, regular urinalysis tests to monitor smoking rates in female volunteer soldiers are more reliable. In addition, because self-reporting underestimated the smoking rate by 7.0%, subsequent studies should be conducted to further understand the factors that cause female volunteer soldiers to engage in inaccurate self-reporting. Since smoking is allowed in the Taiwanese military. Therefore, the results of this study may be generalized to non-military young women in Asia.

## Data Availability

The data presented in this study are available on request from the corresponding author. The data are not publicly available due to privacy.
